# Supplementary material for: βH‐spectrin is required for ratcheting apical pulsatile constrictions during tissue invagination
Source: EMBO Rep. 2020 Jun 26;21(8):e49858. doi: 10.15252/embr.201949858 (PMC7403717; doi:10.15252/embr.201949858)
Supplement: Supplementary file 1 — Expanded View Figures PDF [file EMBR-21-e49858-s001.pdf]

Expanded View Figures

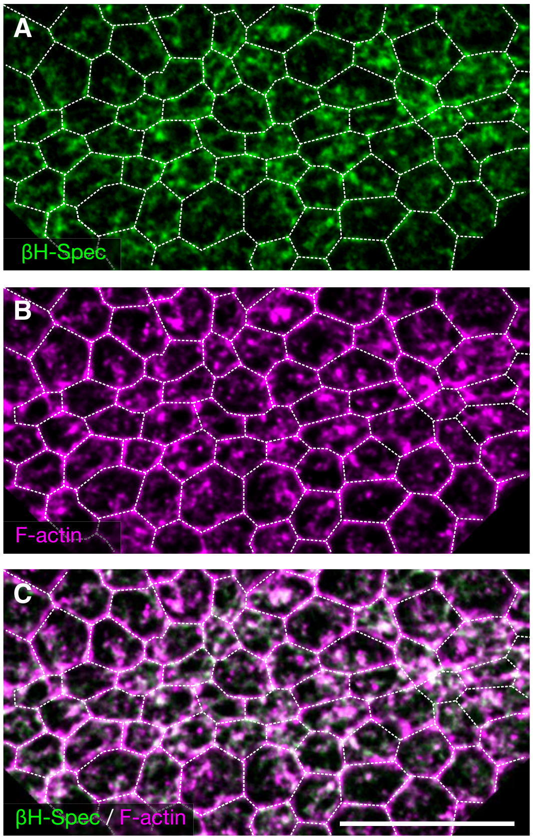

Figure EV1. βH-spectrin co-localizes with F-actin at the apical surface during ventral furrow formation.

A–C Surface projection of the apical cell surface of a *Drosophila* embryo during ventral furrow formation co-stained for F-actin using phalloidin (A) and mVenus::βH-spectrin using FluoTag®-X4 anti-GFP (B). Panel (C) shows a merge of the phalloidin (magenta) and βH-spectrin (green) staining (co-localization analysis: Pearson's R value > 0.7). White dashed lines indicate the cell boundaries based on the phalloidin staining of sub-apical confocal sections. Scale bars: 20 μm.

© 2020 European Molecular Biology Laboratory

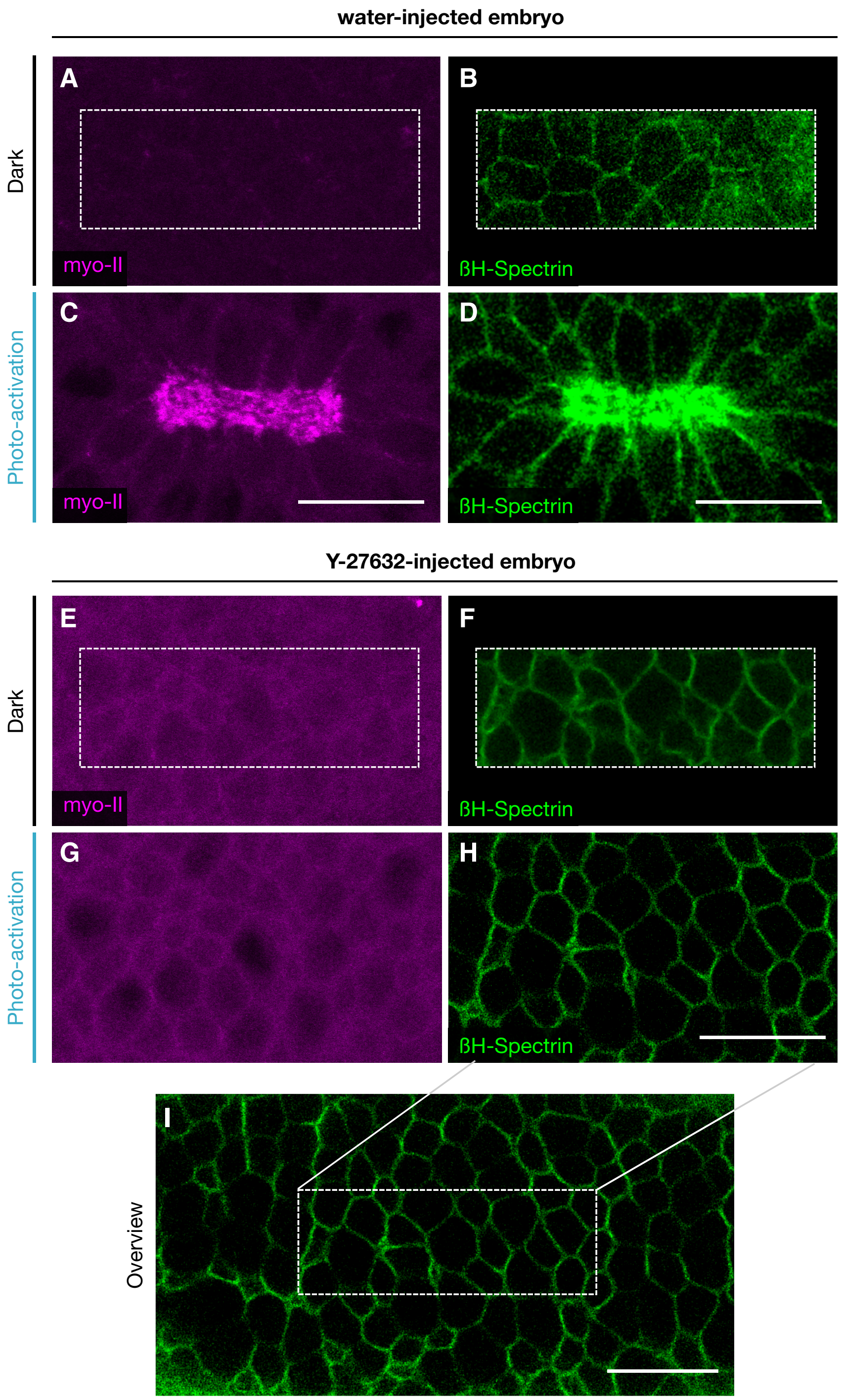

Figure EV2. βH-spectrin upregulation depends on ROCK activity.

A–I Confocal or two-photon microscopy images of *Drosophila* embryos co-expressing the optogenetic module to photo-activate Rho signaling (related to Fig 3C), the myosin-II probe Sqh::mCherry (magenta, A, C, E, G) and mVenus::βH-spectrin (green, B, D, F, H, I). A subset of cells (dashed boxes in A, B and E, F) of the dorsal tissue was photo-activated at the apical surface in embryos previously injected with water (A–D) or the ROCK inhibitor Y-27632 (E–I). The Sqh::mCherry and mVenus::βH-spectrin signal at the apical cell surface was recorded before photo-activation (A, B and E, F) and 1.5 min after photo-activation (C, D and G–I). Panel (I) shows an overview of the mVenus::βH-spectrin signal after photo-activation of the region indicated by a white dashed box. While in water-injected embryos, myosin-II and βH-spectrin levels increased and the cells constricted, in Y-27632-injected embryos their level did not change and the cells did not constrict. Scale bars: 20 µm.

 © 2020 European Molecular Biology Laboratory

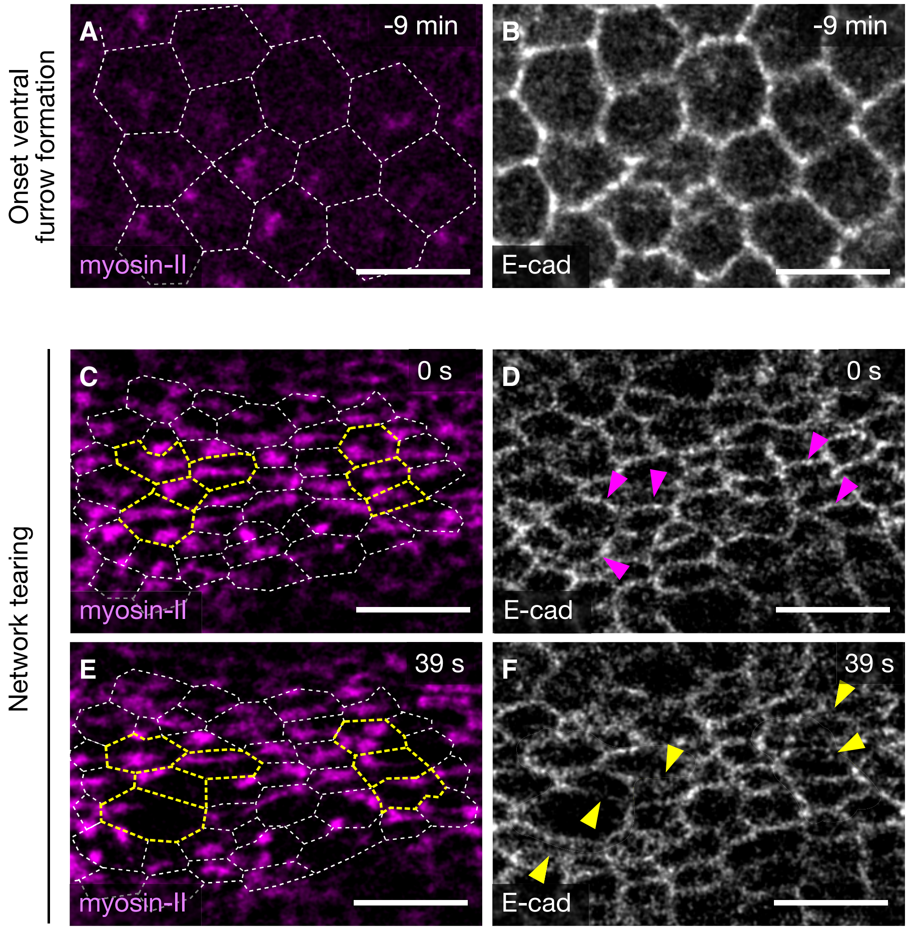

Figure EV3. Adherens junctions dissipate locally as the apical actomyosin network breaks in βH-spectrin knockdown embryos.

A–F Confocal images of the apical surface of a βH-spectrin knockdown embryo co-expressing the myosin-II probe Sqh::mCherry (magenta, A, C, E) and E-cadherin::mNeonGreen (gray, B, D, F). Segmented cell boundaries, generated based on the sub-apical E-cadherin::mNeonGreen signal, are displayed as dashed white lines and overlaid with the Sqh::mCherry signal in A, C, E. At the onset of ventral furrow formation, adherens junctions form normally (A, B). (C–F) Cells, in which the actomyosin network teared (yellow dashed outlines in C, E), show disruption of adherens junctions that were present before the tearing (magenta arrow heads in D) and local dissipation of E-cadherin (yellow arrow heads in F). Scale bars: 10 µm.

© 2020 European Molecular Biology Laboratory

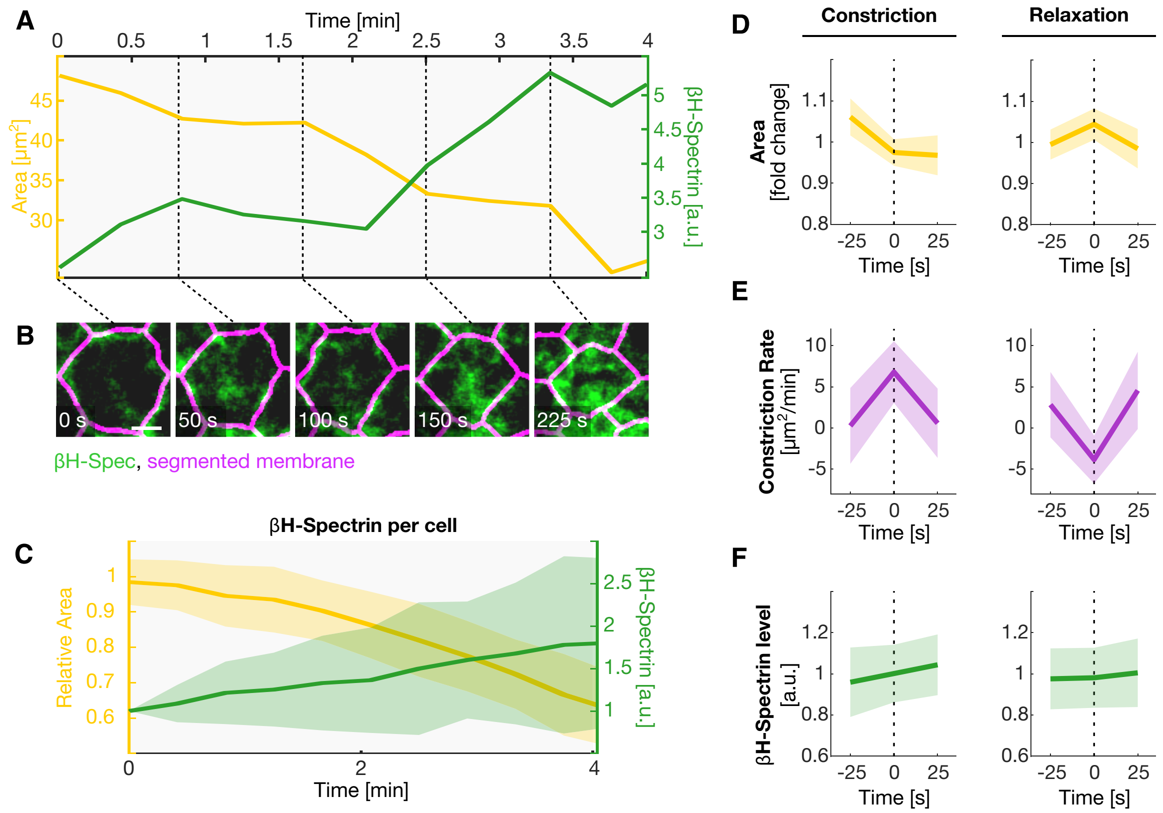

Figure EV4. βH-spectrin accumulates progressively at the apical surface during ventral furrow formation.

A Quantification of cell area (yellow, left axis) and the level of βH-spectrin (green, right axis) in a single cell of a *Drosophila* embryo during ventral furrow formation.

B Still frames of a confocal movie showing apical βH-spectrin (green) and segmented cell boundaries (magenta), generated based on the co-expressed GAP43::mCherry membrane marker, in the cell analyzed in (A). Dashed black lines map the still frames to the respective time points in (A). Scale bar: 2.5 µm.

C Quantification of the average cell area (yellow, left axis) and the average level of βH-spectrin (green, right axis) for n = 52 cells (3 embryos).

D, F Graphs analyzing the average behavior of the cell area (D), constriction rate (E) and βH-spectrin level (F) around constriction peaks (left) and expansion peaks (right). Peaks in the constriction rate (n = 236 peaks) or expansion rate (n = 174 peaks) were identified, respectively, and the cell area, constriction rate, and βH-spectrin level were averaged for all pulsation peaks in a time window between 25 s before and after the individual peaks. βH-spectrin levels increased during constriction peaks and were stable during cell expansion.

Data information: (C–F) In each graph, solid lines represent the mean values and the semi-transparent region the standard deviation.

 © 2020 European Molecular Biology Laboratory

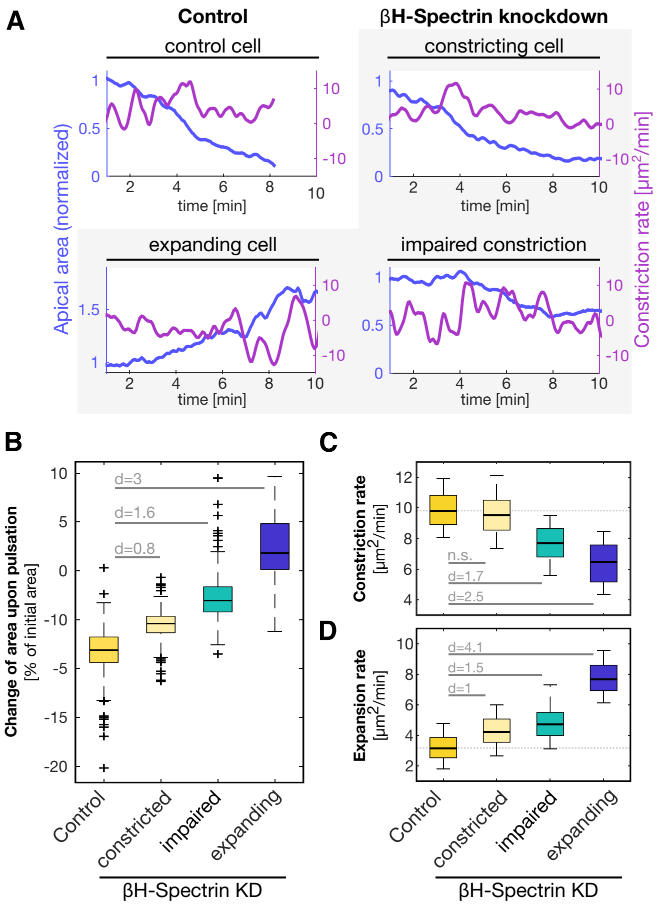

Figure EV5. Pulsations of the apical cell surface in βH-spectrin knockdown embryos are not ratcheted.

A Graphs showing the apical area (on the left *y*-axis, purple) and the corresponding constriction rates (on the right *y*-axis, pink) of a single control cell (top left) and a single βH-spectrin knockdown cell that constricted (top right), that showed impaired constriction (bottom right), and that expanded (bottom left).

B Boxplot showing quantification of the average difference in apical cell area between consecutive pulsation peaks of control cells (n = 274), and of cells in βH-spectrin knockdown embryos that constricted (n = 192), of cells that showed impaired constriction (n = 168), and of cells that expanded (n = 23). The values are presented as percentage of initial cell area. Negative values indicate that the cell area incrementally decreased upon pulsation (ratchet), and values around zero indicate that the cell area relaxed back to the initial size (non-ratchet). ANOVA result (all data points): $F(3,769)$ = 381.5, P = 1e-151; Cohen's d for all comparisons: $0.8 < d \leq 3$.

C, D Boxplot quantifying the rate (change of area per time) of constriction peaks (C) and of expansion peaks (D) in control cells and cells in βH-spectrin knockdown embryos that constricted, showed impaired constriction and expanded. While the mean constriction rate between control cells and βH-spectrin knockdown cells with deficient constriction changed from 9.9 ± 1.1 μm^2/min to 7.5 ± 1.2 μm^2/min (student's *t*-test: P = 4.9e-28; Cohen's d = 1.4) representing a change of −24%, the expansion rate changed from 3.2 ± 0.8 μm^2/min to 5.1 ± 1.4 μm^2/min (student's *t*-test: P = 1.3e-40; Cohen's d = 1.4) representing a change of 60%. Number of sampled cells is equal to (B). (C) ANOVA result (all data points): $F(3,556)$ = 162.1, P = 1.7e-75; Cohen's $d > 1.7$ between control cells and βH-spectrin knockdown cells with deficient constriction. (D) ANOVA result (all data points): $F(3,537)$ = 179, P = 2e-80. Cohen's $d > 1$ between control cells and βH-spectrin knockdown cells.

Data information: (B–D) Cells in βH-spectrin knockdown embryos were classified with regard to the change in apical area as "constricted" when the area changed to < 50%, as "impaired constriction" when the area changed to a range between > 50% and < 100%, as "expanded" when the area changed to > 100%, and as "deficient constriction" when the area changed to > 50%. For each graph, the central mark, the bottom, and the top edge of each box plot indicate the median, the 25th and 75th percentiles, respectively. Whiskers extend to the most extreme data point and crosses indicate outliers. Due to a big effect size (high sample number), statistical significance was assessed based on Cohen's d. A Cohen's d < 0.5 was considered not significant (n.s.).

© 2020 European Molecular Biology Laboratory
