# Supplementary material for: βH‐spectrin is required for ratcheting apical pulsatile constrictions during tissue invagination
Source: EMBO Rep. 2020 Jun 26;21(8):e49858. doi: 10.15252/embr.201949858 (PMC7403717; doi:10.15252/embr.201949858)
Supplement: Supplementary file 2 — Movie EV1 [file EMBR-21-e49858-s002.zip › EMBOR-2019-49858V2_MovieEV1.docx]

**Movie EV1. βH-spectrin is enriched at the apical surface of ventral mesodermal cells during ventral furrow invagination.** Movie showing a *Drosophila* embryo expressing endogenously tagged mVenus::βH-spectrin visualized using two-photon microscopy from the end of cellularization to the end of ventral furrow formation. The embryo was mounted with the posterior side facing the objective. Scale bar, 50 μm.
