# Supplementary material for: βH‐spectrin is required for ratcheting apical pulsatile constrictions during tissue invagination
Source: EMBO Rep. 2020 Jun 26;21(8):e49858. doi: 10.15252/embr.201949858 (PMC7403717; doi:10.15252/embr.201949858)
Supplement: Supplementary file 3 — Movie EV2 [file EMBR-21-e49858-s003.zip › EMBOR-2019-49858V2_MovieEV2.docx]

**Movie EV2. βH-spectrin accumulates at the medio-apical surface and forms a supracellular network along the ventral mesodermal cells.** Confocal microscopy movie showing the apical surface of ventral cells of a Drosophila embryo expressing endogenously tagged mVenus::βH-spectrin (green) and the membrane marker GAP43::mCherry (magenta) during ventral furrow invagination. Scale bar, 20μm.
