# Supplementary material for: βH‐spectrin is required for ratcheting apical pulsatile constrictions during tissue invagination
Source: EMBO Rep. 2020 Jun 26;21(8):e49858. doi: 10.15252/embr.201949858 (PMC7403717; doi:10.15252/embr.201949858)
Supplement: Supplementary file 4 — Movie EV3 [file EMBR-21-e49858-s004.zip › EMBOR-2019-49858V2_MovieEV3.docx]

**Movie EV3. βH-spectrin is required for coordinated apical constrictions.** Concatenated sequence of four confocal microscopy movies showing the apical cell surface of mesodermal cells of *Drosophila* embryos expressing the membrane marker GAP43::mCherry (gray; displayed with inverted color index) during ventral furrow formation. In order of appearance, the movie shows a control embryo, followed by three βH-spectrin knock-down embryos categorized as minor phenotype, severe phenotype with tissue invagination and severe phenotype without tissue invagination. Scale bars, 20 μm.
