# Supplementary material for: βH‐spectrin is required for ratcheting apical pulsatile constrictions during tissue invagination
Source: EMBO Rep. 2020 Jun 26;21(8):e49858. doi: 10.15252/embr.201949858 (PMC7403717; doi:10.15252/embr.201949858)
Supplement: Supplementary file 5 — Movie EV4 [file EMBR-21-e49858-s005.zip › EMBOR-2019-49858V2_MovieEV4.docx]

**Movie EV4. Medioapical localization of βH-spectrin depends on the activity of the mesoderm-specific transcription factor Twist.** Concatenated sequence of two confocal microscopy movies showing the apical surface of the ventral tissue of *Drosophila* embryos expressing endogenously tagged mVenus::βH-spectrin (green) during ventral furrow formation. First a wild type control embryo is shown, followed by a *twist* mutant embryo. Scale bars, 20 μm.
