# Supplementary material for: βH‐spectrin is required for ratcheting apical pulsatile constrictions during tissue invagination
Source: EMBO Rep. 2020 Jun 26;21(8):e49858. doi: 10.15252/embr.201949858 (PMC7403717; doi:10.15252/embr.201949858)
Supplement: Supplementary file 6 — Movie EV5 [file EMBR-21-e49858-s006.zip › EMBOR-2019-49858V2_MovieEV5.docx]

**Movie EV5. Stabilization of the pulsatile apical actomyosin network and junctional integrity depends on βH-spectrin.** Three confocal microscopy movies showing the apical surface of the ventral tissue of *Drosophila* embryos expressing the myosin-II marker Sqh::mCherry (green) and E-cadherin::mNeonGreen (magenta) during ventral furrow formation. At the top a control embryo is shown, in the center a βH-spectrin knock-down embryo with severe phenotype undergoing tissue invagination and at the bottom a βH-spectrin knock-down embryo with severe phenotype not undergoing tissue invagination are shown. Scale bars, 20 μm.
