# Supplementary material for: βH‐spectrin is required for ratcheting apical pulsatile constrictions during tissue invagination
Source: EMBO Rep. 2020 Jun 26;21(8):e49858. doi: 10.15252/embr.201949858 (PMC7403717; doi:10.15252/embr.201949858)
Supplement: Supplementary file 7 — Movie EV6 [file EMBR-21-e49858-s007.zip › EMBOR-2019-49858V2_MovieEV6.docx]

**Movie EV6. Apical F-actin network organization during ventral furrow formation depends on βH-spectrin.** Concatenated sequence of two confocal microscopy movies showing the apical surface of the ventral tissue of *Drosophila* embryos expressing the F-actin probe LifeAct::mNeonGreen (green) and the membrane marker GAP43::mCherry during ventral furrow formation. First a wild type control embryo is shown, followed by a βH-spectrin knock-down embryo. Scale bars, 20 μm.
